# Supplementary material for: Current Use of Oral Anticoagulation Therapy in Elderly Patients with Atrial Fibrillation: Results from an Italian Multicenter Prospective Study—The ISNEP Study
Source: J Pers Med. 2022 Aug 31;12(9):1419. doi: 10.3390/jpm12091419 (PMC9505177; doi:10.3390/jpm12091419)
Supplement: Supplementary file 1 [file jpm-12-01419-s001.zip › jpm-1802624-supplementary.pdf]

Supplementary Material Table S1 – LIST OF ISNEP STUDY INVESTIGATORS

**REGION of Italy**	**City**	**Surname**	**Name**
Campania	Castel Volturno, Caserta	Marullo	Luciano
Campania	Maddaloni, Caserta	Severino	Salvatore
Campania	Acerra, Naples	Sidiropulos	Milena
LOMBARDIA	Gardone Riviera, Brescia	Chimini	Claudio
LOMBARDIA	Milan	Colombo	Chiara
MARCHE	Ancona	Antonicelli	Roberto
MARCHE	Jesi, Ancona	Boria	Cesare
Piemonte	Piossasco, Turin	Micanti	Alex
SICILIA	Palermo	Rebulla	Eduardo
SICILIA	Ragusa	Miano	Marco
TOSCANA	Florence	Bamoshmoosh	Mohamed
